# *AD Course Map* charts Alzheimer’s disease progression

**DOI:** 10.1038/s41598-021-87434-1

**Published:** 2021-04-13

**Authors:** Igor Koval, Alexandre Bône, Maxime Louis, Thomas Lartigue, Simona Bottani, Arnaud Marcoux, Jorge Samper-González, Ninon Burgos, Benjamin Charlier, Anne Bertrand, Stéphane Epelbaum, Olivier Colliot, Stéphanie Allassonnière, Stanley Durrleman

**Affiliations:** 1 Institut du Cerveau et de la Moelle épinière (ICM) & Inserm, U 1127 & CNRS, UMR 7225, Sorbonne Université, 75013 Paris, France; 2 Inria, Aramis project-team, Paris, France; 3 AP-HP, Hôpital de la Pitié Salpêtrière, Paris, France; 4 Centre de Recherche des Cordeliers, Université Paris Descartes, Paris, France; 5 Centre de Mathématiques Appliquées, Ecole Polytechnique, Palaiseau, France; 6 Laboratoire Alexandre Grotendieck, Université de Montpellier, Montpellier, France

**Keywords:** Computational science, Computer science

## Abstract

Alzheimer’s disease (AD) is characterized by the progressive alterations seen in brain images which give rise to the onset of various sets of symptoms. The variability in the dynamics of changes in both brain images and cognitive impairments remains poorly understood. This paper introduces AD Course Map a spatiotemporal atlas of Alzheimer’s disease progression. It summarizes the variability in the progression of a series of neuropsychological assessments, the propagation of hypometabolism and cortical thinning across brain regions and the deformation of the shape of the hippocampus. The analysis of these variations highlights strong genetic determinants for the progression, like possible compensatory mechanisms at play during disease progression. AD Course Map also predicts the patient’s cognitive decline with a better accuracy than the 56 methods benchmarked in the open challenge TADPOLE. Finally, AD Course Map is used to simulate cohorts of virtual patients developing Alzheimer’s disease. AD Course Map offers therefore new tools for exploring the progression of AD and personalizing patients care.

## Introduction

Alzheimer’s disease (AD) is an extraordinarily complex disease. Its effects on the brain are visible on multiple radiological examinations. Their consequences on the cognition and behavior are evaluated by a series neuro-psychological assessments. Adding to this complexity, the disease develops during decades of life, the progression is not linear, and the timing among radiological and clinical signs greatly varies across individuals.

For all these reasons, it is difficult for the clinicians to predict how the disease will progress in each patient and to forecast how the progression of one patient will differ from another one. This difficulty hampers the identification of the optimal moment to test a therapeutic intervention and a fine-grained description of how the drug modifies the different aspects of disease progression.

In this paper, we propose a system to position any patient at any time-point in a map of disease progression, called AD Course Map. AD Course Map summarizes the range of likely trajectories followed by patients developing AD. The positioning includes the identification a precise disease stage and a patient specific trajectory showing how the patient’s data will change in the coming years. The positioning of several subjects opens up the possibility to explore the variability of disease manifestation via a small number of interpretable parameters.

We construct AD Course Map using a statistical learning algorithm and a longitudinal multimodal observational data set. The statistical analysis of longitudinal data is challenging because standard methods do not account for age at onset and pace of progression as possible co-founding factors^1^. In fact, data from different subjects may differ at least two reasons. First, data vary because the disease progresses with different dynamics. Even two patients showing a very typical form of the disease with the same sequence of radiological and clinical signs may develop it at a different age and at a different pace. Even if they follow the same trajectory, the data of these two patients taken at the same age still differ: patients are not at the same disease stage. Second, data vary because the presentation of the disease varies according to patients. One patient may show a predominantly amnesic form of the disease, while another one show primarily attention deficits. Therefore, even if both patients are observed at the same disease stage, the assessments of their memory and attention capacity still differ. The statistical analysis should disentangle such phenotypic differences from differences in the dynamics of progression.

Describing the differences in the dynamics of progression has been tackled in previous disease modeling techniques using functions that map the actual age of the patient to a stage of progression. These functions have been introduced as time-warps^2,3^ and have been included in latent time models^4,5^. Several statistical models assume that all patients follow the same trajectory but with a different dynamics^6–8^. Data take always the same values but not at the same age. These approaches ignore possible differences in disease presentation. Conversely, event-based models^9,10^ explores how the temporal sequence of events varies, the event being defined as the moment when a biomarker becomes abnormal. Nevertheless, this approach does not link the temporal sequence with the age of the patient.

Combining dynamic and phenotypic variations in a statistical model is difficult because their effects may be confounded. Disease course mapping is a statistical technique which allows the decomposition of these two types of variations seen in a longitudinal data set. It builds on abstract geometric principles so that it is not specific to particular data^11,12^. It combines the concept of time-warp with translations of curves.

This technique extends the concept of atlas in neuroimaging to locate anatomical structures in brain images independently of their variations in size and overall shape of the brain. Each anatomical structure has its own set of (x,y,z) coordinate in a reference image of the brain. The reference image is transformed so that it superimposes with the image of the subject. This transformation puts into correspondence each (x,y,z) coordinate of the atlas to a point in the image of the subject. It locates therefore the anatomical structures in the subject’s brain. If one repeats this procedure for a set of representative subjects, the transformations applied to the atlas image describe the variability in size and shape of the anatomical structures within the population.

**Figure 1:**
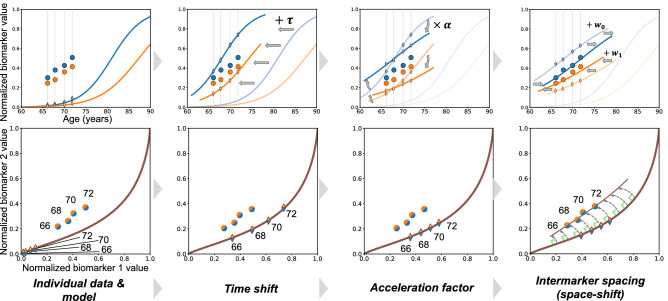
Disease course mapping for two biomarker data. Top left panel: the model as plain curves and repeated data of one subject. x-axis is age in years and y-axis is the normalized values of the biomarkers. Top row, three left panels: the three operations used to mapping the model to the individual data: the time-shift translates the curves, the acceleration factor scales the abscissa, the space-shifts change the time interval between both curves. Bottom row shows another representation of the *same* data as parametric curves. Panels plot the values of one biomarker versus the other one, time being the parameter. Data fall within a unit square, which is a particular case of a Riemannian manifold. The model is a geodesic curve and the transformed curve is a change in the parametric representation of the geodesic followed by exp-parallelisation, a generalisation of translation on manifolds. This construction for two biomarker data extends therefore to any kind of data on a Riemannian manifold.

Disease course mapping extends this concept for the progression of a series of biomarkers. The atlas takes the form a series of progression curves for each biomarker where the time axis represents a disease stage, which we call here an Alzheimer Age (AA). Subject’s data take the form the biomarkers measurements at several time-points. The transformation of model to the subject’s data consist in computing (1) a time-shift, which translates the model along the time axis to accommodate for changes in age at disease onset, (2) an acceleration factor, which scales the time interval to accommodate for differences in speed of progression, and (3) intermarker spacings, which translate each biomarker differently to accommodate for differences in the timing and ordering among biomarkers. The first two parameters define a time-warp function that maps the actual age of the subject to his AA. This function therefore changes the dynamics of progression but not the trajectory. In turn, inter-marker spacings shift the patient trajectory to account for phenotypic differences across subjects, as illustrated in Figure 1.

Disease course mapping is not specific to biomarker data. It further extends to the case where data are images or meshes of anatomical structures. The reference scenario is replaced by a scenario of temporal changes of image intensities or a scenario of continuous deformation of a shape respectively. Time-shifts and acceleration factor plays the same role of adjusting for the dynamics of change. They map the actual age of the subject at each visit to its Alzheimer Age that is a time-point in the reference scenario. Intermarker spacing adjust for the spatiotemporal pattern of changes across brain regions or for the shape of the structure respectively. In this setting intermarker spacing takes the more general name of space-shift.

We use here a longitudinal data sets of patients who were diagnosed with Alzheimer’s disease at some point of the study to reconstruct the natural history of the disease. We construct an AD Course Map showing the reduction in glucose consumption across brain regions, the thinning of the cerebral cortex, the deformation of the hippocampus due to atrophy, and the progressive onset of cognitive deficit from the preclinical to the clinical phase of the disease.

We analyse the distribution of the parameters that adjust the reference scenario of disease progression to each subject’s data. This statistical analysis aims to highlight how genetic, biological or environmental factors change disease progression profiles. Once the map is built, we position new individuals into the map and then read how data will change in the coming years. Eventually, we build a virtual cohort of patients developing AD by sampling random trajectories in the map.

## Results

### AD Course Map: a multimodal atlas of disease progression

**Figure 2:**
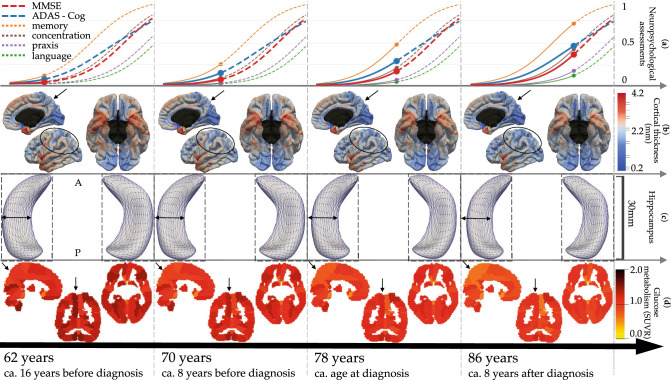
Normative models of Alzheimer’s disease progression shown at 4 Alzheimer Age with estimated time until/from diagnosis. Bottom to top rows show alteration of brain glucose metabolism, hippocampus atrophy, cortical thinning and onset of cognitive decline. Black arrows and ellipses indicate some areas of great changes. Images were obtained using the freely available software FSLeyes v.0.22.6 (https://fsl.fmrib.ox.ac.uk/fsl/fslwiki/FSLeyes) and Paraview v.5.2.0 (www.paraview.org).

We estimated AD Course Map using multimodal longitudinal data from subjects covering the pre-clinical to the clinical stage of the disease (see Methods). Figure 2 shows at four representative Alzheimer ages (AA) the normative scenario of cognitive decline (first row), spatiotemporal propagation of cortical thinning across cortical regions (second row), progressive deformation of the hippocampus in both hemispheres due to atrophy (third row), and spatiotemporal progression hypometabolism across brain regions (fourth row). AD Course Map may be visualised at a fine temporal resolution in the form of an interactive visualisation at the website: www.digital-brain.org.

We found that the AA at the time of diagnosis is of 78 $(\pm 5.6)$ years. The model represented in Fig. 2 encompasses therefore 16 years before diagnosis and 8 years after. It has been shown that Alzheimer’s disease diagnosis occurs when the ADAS-Cog is comprised between 18.6 and 28.9 (i.e. between 0.21 and 0.34 on the normalised scale)^13^, which is reached in the AD Course Map for an AA between 74 and 80 years. Similarly, the diagnosis usually occurs for a MMSE score comprised between 27 and 23 (i.e. 0.1 and 0.23 on the normalised scale)^14^, which occurs in the AD Course Map for an AA between 74 and 81 years. These age ranges are compatible with our estimation of the diagnosis occurring at an AA of 78 $(\pm 5.6)$ years.

AD Course Map shows a typical sequence of cognitive impairments starting with memory, followed by concentration 9.6 ($\pm 1.54$) years after, praxis 9.8 ($\pm 1.73$) years after, and finally language 3.3 ($\pm 2.65$) years after. The greatest alterations of glucose hypometabolism are found in the precuneus^15–17^, prefrontal areas^18^ and the parahippocampal region^19^. Cortical atrophy also occurs in typical regions such as enthorinal cortex, hippocampal gyrus, temporal pole and fusiform gyrus^20,21^, cortical association areas^22,23^ and precuneus^24^. As expected, very little atrophy is shown to occur in the occipital lobe and the cingulate gyrus. These structural and functional alterations are in line with previous findings. More surprisingly, the model shows atrophy in the precentral gyrus and the paracentral lobule. Whether these regions are affected by cortical thinning due to Alzheimer’s disease is still a debated question^25^. We found that the level of noise in these regions is one of the largest, a fact that may explain inconsistencies across studies.

#### AD Course Map fits data with an error of the order of the measurement uncertainty

AD Course Map estimates not only a typical progression of clinical and imaging data, but also a range of possible trajectories. These trajectories derive from the typical scenario by adjusting the age at onset, pace of progression, appearance of the images, the shape of meshes, and the temporal spacing among clinical assessments.

After model calibration, each subject is positioned on one of these possible trajectories. The positioning amounts to the estimation of subject-specific time-shift, acceleration factor and a series of space-shift for each modality. The space-shifts determine the subject-specific trajectory. The time-shift and acceleration factor allow the computation of the Alzheimer Age of the subject, and therefore locate one point on the trajectory at each visit. We measure now to which extend the data at these points on the trajectory are similar to the observed data. We call the difference the *reconstruction error*.

**Figure 3:**
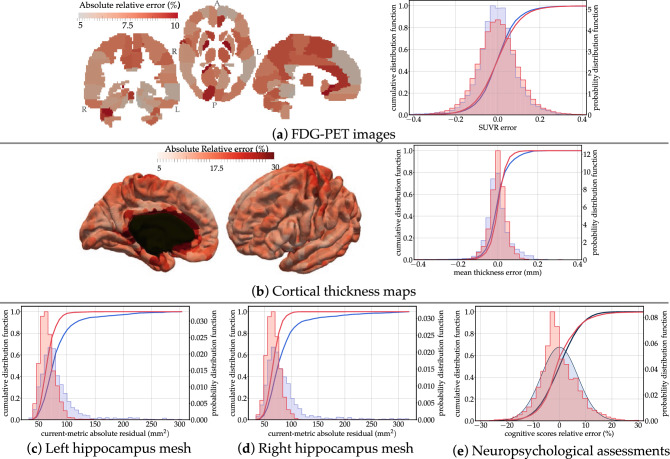
Distributions of reconstruction errors. The empirical distribution of errors (red) is superimposed with the estimated distribution of test / re-test differences (in blue). The absolute relative error is shown in every brain region for FDG-PET images and cortical thickness maps. Mean and standard errors are given in Supplementary Table S1. Images were obtained using the freely available software FSLeyes v.0.22.6 (https://fsl.fmrib.ox.ac.uk/fsl/fslwiki/FSLeyes) and Paraview v.5.2.0 (www.paraview.org).

Figure 3 shows the distribution of the reconstruction errors for all visits from all subjects. We notice that the reconstruction errors in brain regions are not evenly distributed. For PET data, the largest errors are found mostly in smaller regions. For cortical thickness, larger errors are found at the boundary of the mesh with the corpus callosum, mostly due to interpolation errors. These errors are much smaller than the best possible image resolution of $1\,\text {mm}$ isotropic, thus making these reconstructions at sub-voxel precision. Reconstruction error for the hippocampus is reported in terms of the current distance^26^, which is measure of difference in shape between two meshes. It is robust to mesh imperfection, spikes and holes. Errors in terms of volume are reported in Supplementary Figure S1.

We further evaluate how good is the goodness-of-fit by estimating the uncertainty of the measurements due to acquisition and processing. Imaging data are subject to variations in the acquisition protocol and experimental conditions such as slight motion of the subject in the scanner. Clinical assessments are subject to inter-rater variability and subject’s condition. Research protocols tend to reduce these variations as much as possible. Nevertheless, data are known only within an error margin which is difficult to evaluate. For MRI data, we estimate this uncertainty by measuring differences between test and re-test MRI sessions, two images acquired on the same subject one right after the other. For PET data, there is no such test / re-test data. As a proxy, we measured the difference between the baseline and follow-up image after 18 months for amyloid negative cognitively normal subjects. It is an over-estimation of the measurement uncertainty as this proxy neglects the effect of normal aging on the change in FDG uptake. Nevertheless, studies have not shown statistically significant longitudinal changes in FDG uptake over such a short period of time for normal ageing individuals^27,28^. Another study reports significant longitudinal changes over an average period of 7.8 years in the anterior and posterior cingulate cortex with annual rates of change of 0.58% and 0.57% respectively^29^. For the neuropsychological assessments, we perform a review of the literature assessing their reproducibility (see Methods). From these estimates, we deduce an empirical distribution of the noise in the data.

Fig. 3 shows the superimposition of the empirical distribution of reconstruction errors with the empirical distribution of the noise for all data types. Overall, the two distributions largely overlap, and the standard error is of the same order than the measurement noise (see Supplementary Table S1). This result shows that our model explains the variance seen in the data up to the noise level. Therefore, for the metric on the data that we used, these results could not be improved without over-fitting.

#### AD Course Map fits new subjects’ data equally well

Once the AD Course Map has been calibrated on a representative data set, we aim to use it to analyse the data of new subjects. We estimate here the accuracy of AD Course Map to fit new subject’s data.

We use a five-fold cross validation procedure. The model is calibrated using 80% of the subjects, and the remaining 20% subjects are then positioned on the map. In the positioning phase, we use the typical scenario of progression and the covariance among subject specific parameters, which were estimated during the calibration phase. For each of the unseen subjects, we only estimate one time-shift, one acceleration factor and a series of space-shifts that minimize the discrepancy between the predicted data and the true subject’s data. We call this discrepancy a *generalisation error*.

Distributions of the generalisation errors are essentially identical with these the reconstruction errors (see Supplementary Fig. S2). Only hippocampus shows a slightly higher generalisation error but still below the noise level estimated with test / re-test data. The goodness-of-fit is therefore equally good whether the subject has been included in the training set or in the test set.

#### AD Course Map calibration is robust against resampling

After the cross-validation procedure, each subject has been used exactly once as a test subject. We consider the subject-specific parameters that were estimated at that time. We compare them with the parameter values that were estimated when calibrating the model on the whole data set, and thus when the considered subject was used in the training set. The difference between the two estimates is small with $r^2$ comprised between 0.93 and 0.99 (see Supplementary Fig. S3).

Eventually, we use the cross-validation procedure to assess the robustness of AD Course Map against resampling. After the procedure, we have estimated five different course maps. Each course map is determined by the parameters of the typical model of progression and the variance of the subject-specific parameters. We compare these parameters estimated using $80\%$ of the subjects with the ones resulting of the calibration of the model on the whole data set. These parameters show very little variations, thus showing the robustness of the estimation algorithm against resampling in the training set (see Supplementary Table S2).

### AD Course Map summarizes the variety of possible disease progression profiles

AD Course Map positions the progression of each subject with respect a reference. The time-shift and acceleration factor explain differences in age at onset and pace of progression. The set of space-shifts explain how different the subject’ trajectory is in terms of image appearance, shape or timing among clinical assessments. Altogether these subject-specific parameters form the coordinates of the subject in a spatiotemporal coordinate system.

The set of these parameters describes the heterogeneity in disease progression profiles in the studied population. This analysis allows us to disentangle the variations in the dynamics of progression (how early or late is the disease onset of the subject? how fast or slow his progression?) from the variation in the disease presentation (does the subject’s brain exhibit a particular shape, does it show a different ordering in the onset of the symptoms or in the functional alterations across brain regions?)

#### AD Course Map highlights possible compensatory mechanisms

For each modality, we perform a multivariate linear regression between each individual parameters and genetic, biological and environmental factors: sex, APOE-$\varepsilon$4 genotype, presence of amyloidosis, marital status and education level. We identify statistically significant associations using a two tailed t-test at $5\%$ significance level corrected for multiple comparisons with the false discovery rate method (see Methods). Note that in this section, we discard subjects without assessments of amyloidosis (see Supplementary Table S3 for corresponding number of samples).

**Table 1:** Significant associations of individual parameters with genetic, biological and environmental factors: effect sizes, confidence intervals at $95\%$, and adjusted p-values. Only adjusted p-values below $5\%$ significance level are shown. Time-shifts are in months, other quantities have no units. Directions of space-shift are not signed. The figures on the top of the column “hippocampal atrophy” reads: “atrophy of the left hippocampus progresses 1.27 times faster in women than in men, starts 33.6 months earlier, and the hippocampus shape is significantly different between men and women regardless of the disease stage”.

			Hypometabolism	Hippocampus atrophy (MRI)		Cortical thinning	cognitive decline
			(FDG-PET)	:Left hemisphere	Right hemisphere	(MRI)	(ADAS+MMSE)
Genetic	$$\begin{array}{l}\hbox {sex}\\ \hbox {female vs. male}\end{array}$$	Accel. factor		$$\times 1.27 \quad \begin{array}{c}\hbox {CI}=[1.11, 1.45] \\ p = 2.26\hbox {e}{-}3^{**}\end{array}$$	$$\times 1.26 \quad \begin{array}{c}{\hbox {CI}=[1.08, 1.45]} \\ {p = 6.15-3^{**}}\end{array}$$		$$\times 1.46 \quad \begin{array}{c} {\hbox {CI}=[1.10, 1.92}] \\ {p = 8.42\hbox {e}{-}3^{**}}\end{array}$$
		Time-shift		$$-33.6\quad \begin{array}{c} {\hbox {CI}=[-55.8, -11.6]} \\ {p = 3.71\hbox {e}{-}3^{**}}\end{array}$$	$$-29.0\quad \begin{array}{c}{\hbox {CI}=[-53.0, -4.91]} \\ {p = 2.31\hbox {e}{-}2^{*}}\end{array}$$		$$-36.8\quad \begin{array}{c}{\hbox {CI}=[-62.0, -11.6]} \\ {p = 4.48\hbox {e}{-}3^{**}}\end{array}$$
		Space-shift		$$\pm \, 0.55\quad \begin{array}{c}{\hbox {CI}=[0.28, 0.82]} \\ {p = 4.00\hbox {e}{-}4^{***}}\end{array}$$	$$\pm \, 0.60\quad \begin{array}{c}{\hbox {CI}=[0.34, 0.86]} \\ {p = 3.89\hbox {e}{-}5^{****}}\end{array}$$	$$\pm \, 0.48\quad \begin{array}{c}{\hbox {CI}=[0.22, 0.75]} \\ {p = 2.24\hbox {e}{-}3^{**}}\end{array}$$	
	$$\begin{array}{l}\hbox {APOE-}\varepsilon 4\\ \hbox {carrier vs. non-carrier}\end{array}$$	Accel. factor		$$\times 1.17\quad \begin{array}{c}{\hbox {CI}=[1.02, 1.33]} \\ {p = 2.77\hbox {e}{-}2^{*}}\end{array}$$		$$\times 1.42\quad \begin{array}{c}{\hbox {CI}=[1.12, 1.82]} \\ {p = 2.17\hbox {e}{-}2^{*}}\end{array}$$	$$\times 1.25\quad \begin{array}{c}{\hbox {CI}=[1.03, 1.51]}\\ {2.17\hbox {e}{-}2^{*}}\end{array}$$
		Time-shift		$$-45.0\quad \begin{array}{c}{\hbox {CI}=[-66.9, -23.2]} \\ {p = 1.57\hbox {e}{-}4^{***}}\end{array}$$	$$-36.8\quad \begin{array}{c}{\hbox {CI}=[-60.5, -13.0]} \\ {p = 4.27\hbox {e}{-}3^{**}}\end{array}$$		
		Space-shift					
Biological	$$\begin{array}{l}\hbox {amyloid}\\ \hbox {positive vs. negative}\end{array}$$	Accel. factor		$$\times 1.18 \quad \begin{array}{c}{\hbox {CI}=[1.06, 1.32]} \\ {p = 8.20\hbox {e}{-}3^{**}}\end{array}$$	$$\times 1.23\quad \begin{array}{c}{\hbox {CI}=[1.09, 1.39]} \\ {p = 4.03\hbox {e}{-}3^{**}}\end{array}$$		
		Time-shift					$$-21.9\quad \begin{array}{c}{\hbox {CI}=[-41.2,-2.5]} \\ {p = 2.70\hbox {e}{-}2^{*}}\end{array}$$
		Space-shift				$$\pm \, 0.28 \quad \begin{array}{c}{\hbox {CI}=[0.05, 0.50]} \\ {p = 2.24\hbox {e}{-}3^{**}}\end{array}$$	
Environmental	$$\begin{array}{l}\hbox {marital}\\ \hbox {married vs. non-married}\end{array}$$	Accel. factor		$$\times 1.25 \quad \begin{array}{c}{\hbox {CI}=[1.07, 1.48]} \\ {p = 1.08\hbox {e}{-}2^{*}}\end{array}$$			
		Time-shift		$$-59.5 \quad \begin{array}{c}{\hbox {CI}=[-86.6, -32.5]} \\ {p = 1.06\hbox {e}{-}4^{***}}\end{array}$$	$$-52.7 \quad \begin{array}{c}{\hbox {CI}=[-82.2, -23.2]} \\ {p = 1.28\hbox {e}{-}3^{**}}\end{array}$$		$$-32.6 \quad \begin{array}{c}{\hbox {CI}=[1.8, 63.3]} \\ {p = 3.78\hbox {e}{-}2^{*}} \end{array}$$
		Space-shift					
	$$\begin{array}{l}\hbox {education}\\ \hbox {nb. of years of education}\end{array}$$	Accel. factor					
		Time-shift		$$-6.04\quad \begin{array}{c}{\hbox {CI}=[-9.67, -2.42]} \\ {p = 1.95\hbox {e}{-}3^{**}}\end{array}$$	$$-7.60\quad \begin{array}{c}{\hbox {CI}=[-11.55, -3.64]} \\ {p = 9.53\hbox {e}{-}4^{***}}\end{array}$$		
		Space-shift					

Our results in Table 1 show the predominant role of genetic factors to explain the heterogeneity in disease progression. In particular, disease progression presents a strong sexual dimorphism for hippocampus atrophy and cognitive decline. In women, the hippocampus atrophy occurs on average 33.6 months earlier and 1.27 times faster than in men. This atrophy translates into an earlier and accelerated cognitive decline which occurs 36.8 months earlier and 1.46 times faster in women than in men.

By contrast, APOE-$\varepsilon$4 carriers also exhibit earlier and more pronounced alterations of their hippocampus (45 months ealier and 1.17 times faster in carriers compared to non-carriers), but this effect is, to some extend, alleviated in the onset of cognitive decline which does not occur earlier in carriers than in non-carriers. It is as if brain plasticity compensates for the advance of almost 3 years in hippocampal atrophy, but that once the compensation is made, cognitive decline still manifests itself at a faster rate than in subjects without the mutation.

The absence of associations between cofactors and profiles of hypometabolism may be explained by the fact that focal effects on specific brain areas may be diluted in non-specific regions of interest^30^. Except in four occasions, we found associations with parameters that modulate the dynamics of disease progression, not its presentation. This fact suggests that previous findings showing associations between genetic variants and hypometabolism might be due to the comparison of subjects at different ages or disease stages^30,31^. Age is a poor proxy of disease progression. Time from/to diagnosis alone does not allow to correct for variations in pace of progression. By contrast, we propose here a method to distinguish dynamic from phenotypic variations.

#### AD Course Map exhibits complex interplay between structural and functional alterations and cognitive decline

We construct a graph of a conditional correlations between all variables of all modalities. The statistically significant conditional correlations are represented in Figure 6 where three variables per modality are shown: pace of disease progression (e.g. acceleration factors), delay with respect to onset (e.g. time-shift), and pattern (e.g. space-shifts represented here as a single variable for the sake of simplicity).

Interestingly, the vast majority of significant conditional correlations are found among variables of the same type across modalities, and not among different variables within the same modality. It means that the three aspects of disease progression: pace of progression, age at onset and disease presentation are mostly independent of each other.

Cognitive decline is the consequence of the accumulation of lesions in the brain. Bearing this in mind, this graph confirms that the order and timing of the decline of the different cognitive functions is determined by the order and timing of the brain regions affected by neurodegeneration^32^. It further shows that the age at which these cognitive changes occur is associated partly to the age at which hippocampus atrophy occur, and not to the age at which hypometabolism and cortical atrophy starts. These weak to no association may be interpreted again as the consequence of possible compensatory mechanisms for a part of the patients.

We do not find association between pace of progression and age at onset, except for cortical atrophy. Studies reported that some early form of the disease are associated with more rapid progression^33,34^, while others do not find any association^35,36^. Such association studies often use an arbitrary threshold on the diagnosis age to divide early from late onset AD patients. Within each group, a linear rate of change is computed, thus assuming that cognition declines in a linear fashion at all stages. By contrast, our method accounts for a non-linear dynamic of change. It positions patients along a continuous disease progression timeline without the need to cluster patients into arbitrary categories.

### AD Course Map predicts the future progression of new subjects

We evaluate the ability of AD Course Map to predict the disease progression in the distant future for the subjects at risk of developing AD. For this purpose, we select subjects and visits in the ADNI database based on criteria that can be assessed from present and past visits only, without the need to know the whole disease history of the patients as previously.

We select all the visits of all the subjects in ADNI, which met the following conditions:

- the subject is labeled as Mild Cognitive Impairment at this visit,
- the MMSE of the subject is smaller or equal to 27 at this visit, as a confirmation of the MCI stage,
- the subject is amyloid positive at this visit,
- the sequence of diagnosis labels in the past visits is monotonic, meaning we exclude subjects showing reversion to cognitively normal, or having AD label in the past.

These criteria target patients with MCI due to AD according to NIA-AA criteria^37^, a population of interest for clinical trials^38^.

For each subject, we select the visits for which there is at least one follow-up of the same subject 3 or 4 years later in time. We hide the follow-up visits after the considered visit. We position the subject onto the AD Course Map using subject’s data until the considered visit only. We read the subject’s data at the next hidden visits on the subject’s trajectory. We compare the predicted data at the time-point of the follow-up visits with the data that were hidden. We call this difference a “prediction error” (see Methods).

If the selected subject is not part of the previous training data set, we use the AD Course Map estimated in the previous section. If the selected subject is part of it, we use the AD Course Map that was estimated during the cross-validation procedure, using the fold in which the considered subject was a test subject.

We predict the MMSE and the ADAS-Cog for 136 subjects for the prediction at 3 years, and 80 subjects for the prediction at 4 years. We predict MRI data (cortical thickness maps and hippocampus shape) for 72 subjects at 3 years, and 63 subjects at 4 years. The prediction of the neuropsychological assessments was done using patient data at only one time-point in 36.5% of the cases at 3 years, and 39.1% at 4 years. Prediction of the MRI data used only one visit in 33.3% of the cases at 3 years and 58.9% at 4 years.

**Figure 4:**
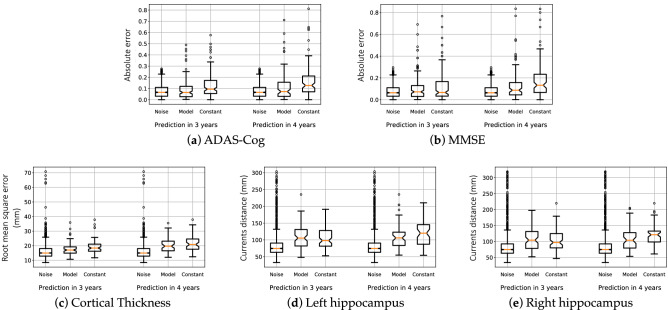
Prediction errors at the individual level 3 and 4 years ahead of time. Box-plots show medians in orange, quartiles, and $95\%$ confidence intervals for three image data and the ADAS-Cog. Distributions of prediction errors are compared with that of the noise and the errors of the constant prediction.

We report in Fig. 4 the error of prediction for the neuro-psychological assessments, the map of the cortical thickness and the shape of the hippocampus of both hemispheres. We compare the prediction errors with the distribution of the noise in the measurements and with the “constant” prediction, which predicts the data will not change in the next 3 or 4 years.

The mean absolute error (MAE) of the prediction of the MMSE is of 3.2 points at 3 years and 4 points at 4 years. These errors are of the same order as the accuracy of the test, which is of about 10%, so 3 points on a scale of 30. For ADAS-Cog, the MAE of 7.6 points at 3 years and 10.1 points at 4 years, where the 10% error range is of 8.5 points.

The error of the constant prediction, though increasing with the time-to-prediction, remains small in comparison with the measurement uncertainty up to 4 years in time. The difference in the median is of 0.001 at 3 years and 0.02 at 4 years for the MMSE normalized on a zero to one scale ($p<0.05$ with a Man-Whitney U test), thus meaning an error smaller than 1 point of MMSE. It has already been shown that neuropsychological assessments, such as the MMSE, lack robustness to track longitudinal changes over short periods of time^39^. These results advocate for longer term predictions, and possibly more sensitive imaging markers^40^.

For MRI derived data, the noise distribution presents a very heavy tail that is due to the large heterogeneity of the image quality and its consequence in data processing, at least using the standard acquisition protocol and processing pipelines of ADNI. The constant prediction does not show such a heavy tail, as images of best quality 3 or 4 years apart show less variability than the test and re-test image acquired the same day. Our model shows steady performance at 3 and 4 years, whereas the constant prediction worsens as time-to-prediction increases.

Comparing such predictions with alternative methods is difficult, as they are often evaluated on different sets of subjects^41,42^. Prediction errors might be easily increased by selecting more stable subjects and calibrating the method to predict little to no changes. For a fair comparison, we re-run our prediction method in the same setting as the TADPOLE challenge and compare our predictions with the 56 competing methods^43^. We estimated another AD Course Map using the TADPOLE training set only and predict future progression of the ADAS-Cog and ventricular volume for the TADPOLE test set. The prediction error of the ADAS-Cog is of $3.70\pm 0.26$(std). It is 21% smaller than the best competing method showing an error of 4.70. The constant prediction yields an MAE of $7.077 \pm 0.352$. Prediction error of the ventricular volume is of $0.412 \pm 0.056$(std), which is the same as the best performing method.

### AD Course Map simulates cohorts of virtual patients

We use AD Course Map to simulate entirely synthetic patients developing Alzheimer’s disease. After model calibration, we estimate the empirical posterior distribution of the subject-specific parameters. We sample then random parameters from this distribution. These parameters are used to transform the reference trajectories into a series of trajectories reproducing the heterogeneity seen in the original population. We pick randomly an arbitrary number of time-points in these trajectories to create a synthetic longitudinal data set. We perform this procedure independently for men and women (see Methods).

To validate such simulations, we aim to replicate the original ADNI data set by picking the same number of time-points and at the same frequency as in the ADNI data set, and by simulating men and women subjects with the same sex ratio. We then compute in the simulated data the regional SUVR, cortical thickness, hippocampus volume and neuro-psychological assessments. We superimpose them with the distributions of the original and reconstructed data.

**Figure 5:**
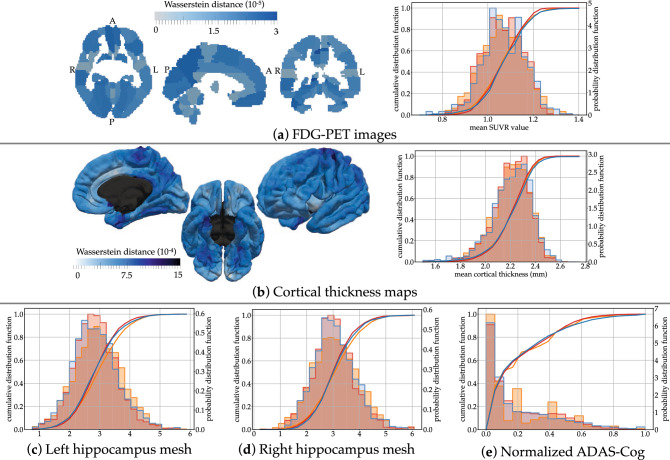
Statistics of the virtual cohort. Superimposition of empirical distributions for simulated data (blue), reconstructed errors (red, as in Fig. 3) and real data (orange). Images were obtained using the freely available software FSLeyes v.0.22.6 (https://fsl.fmrib.ox.ac.uk/fsl/fslwiki/FSLeyes) and Paraview v.5.2.0 (www.paraview.org).

**Figure 6:**
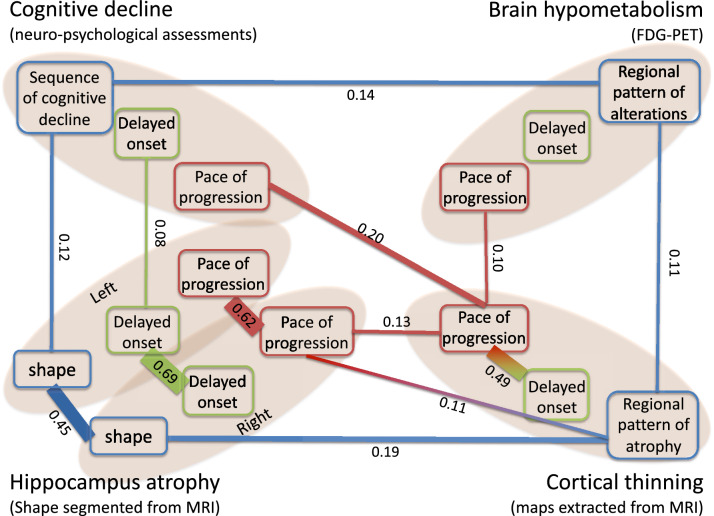
Graph of conditional correlations among individual parameters. An edge is shown between two parameters if there is a significant correlation between them given all other parameters. The width of the edge is proportional to the value of the conditional correlation, which is also reported on the edge. The color of the parameter denotes its type and its position the modality. The image was obtained using the software Microsoft PowerPoint v.16.43.

The superimposition of the distributions in Figure 5 shows that the simulated data closely replicate the reconstructed data for all modalities. For the hippocampus volume, the simulated data have the same bias than the reconstructed data in comparison to the real data. This fact is expected as the simulation reproduces the variability learned by the model. This experiment shows that AD Course Map can be used as a simulator of subjects developing Alzheimer’s disease, which replicates the heterogeneity in disease progression of the studied population.

We have released a virtual cohort of one million of subjects developing AD that is freely available at www.digital-brain.org.

This simulator can be used to arbitrarily increase the number of subjects, number of visits and visits frequency in the training cohort. These data augmentation and resampling techniques are essential to improve the performance of machine learning algorithms. It can also be used to create large validation sets to better evaluate the generalisation errors of such algorithms.

Such a simulator can be seen also as an anonymous replicate of the training cohort, which can therefore be transferred and shared without regulatory constraints in lieu of the data itself. In this way, they allow the comparison and combination of multiple cohorts that would otherwise be very difficult to aggregate. They can thus be used to detect the respective biases of these cohorts, and possibly correct them by simulating patients with a re-balanced disease stage distribution, sex ratio, or ratio of APOE-$\varepsilon$4 carriers for instance. The identification of such biases is essential because they are then found in the predictive systems trained on these cohorts.

## Discussion

AD Course Map charts the possible trajectories of progression to AD. Few and interpretable parameters adjust a reference scenario of progression to the data of a subject. They allowed us to highlight strong sexual dimorphism and possible compensatory mechanisms at play during AD progression.

The personalized subject’s trajectory predicts the future image and clinical data at any time-point in the future. Such predictions are competitive with the state-of-the-art machine learning methods when tested on the same population. The prediction errors are of the order of the measurement uncertainty.

Alternative methods are designed to more directly inform about possible biological mechanisms, such as the propagation of misfolded proteins in the brain^44,45^. Nevertheless, our approach is not specific to certain types of data, and may be applied easily to other therapeutic areas.

One key open question remains the potential use of these disease modeling techniques in clinical decision support systems. The promise of AD Course Map is to target the right patient at the right time. Whether such selection tools eventually lead to more powered clinical trials still needs to be shown.

## Methods

### Data set

We use data from the Alzheimer’s Disease Neuroimaging Initiative (ADNI) to reproduce the natural history of the disease from the pre-clinical to the clinical stage. We thus selected the 322 subjects in the ADNI, ADNI-GO and ADNI-2 databases who were included as cognitively normal or with mild cognitive impairments and who had a confirmed clinical diagnosis of Alzheimer’s disease at a later time-point in the study. Definitions of these categories are as in the ADNI protocol.

Whenever available, we use at each visit:

- regional measurements of standard uptake value ratio (SUVR) of fluorodeoxyglucose (FDG)-positron emission tomography (PET) to build models of hypometabolism across brain regions,
- maps of cortical thickness defined on a mesh of the cortex and extracted from T1-weighted Magnetic Resonance Images (MRI) to build models of cortical thinning,
- surface meshes of the hippocampus of both hemispheres segmented also from T1-weighted MRI to build models of hippocampal atrophy, and
- scores of the Mini-Mental State Examination^46^ (MMSE) and Alzheimer’s Disease Assessment Scale - Cognitive Subscale with 13 items^47,48^ (ADAS-Cog), the latter being divided into four sub-scores assessing memory, language, concentration and praxis, to build models of cognitive decline,

which amounts to 687 visits with PET images, 1993 visits with MRI data and 1235 visits with neuro-psychological assessments (See Supplementary Table S3 for summary statistics).

For each subject, we used the following additional data: age at each visit, sex, marital status, educational level, Apolipoprotein E (ApoE) polymorphism, and presence of amyloidosis. More precisely, we define:

- marital status as: married versus non-married meaning widowed, divorced, or never married;
- educational level as the number of years of education;
- ApoE-$\varepsilon$4 carriership as the presence of at least one allele $\varepsilon$4 of the ApoE gene;
- Amyloid status as positive if one of these conditions was met at one visit at least:
  - a Standard Updake Value ratio (SUVR), normalised by the entire cerebellum, greater than 1.1 in a PET image acquired with Florbetapir (AV-45) compound^49,50^;
  - an average SUVR, normalised by the cerebellum, greater than 1.47 in a PET image with a Pittsburgh compound B (PiB)^50^;
  - a level of beta amyloid 1-42 (A$\beta$42) (measured with the Roche Elecsys assays (see http://www.adni.loni.usc.edu/new-csf-a%CE%B21-42-t-tau-and-p-tau181-biomarkers-results-from-adni-biomarker-core-using-elecsys/) in the cerebrospinal fluid (CSF) lower than 1098 pg/mL^51^;
  unknown if no values of CSF biomarkers and no AV45 or PiB PET images were available at any visit in the ADNI-merge file; and negative otherwise.

### Data pre-processing

MMSE total score, ADAS-cog total score and sub-scores were all normalized to a scale of zero to one, such that normal values correspond to zero and the maximum pathological value to one. Sub-scores were obtained by adding the following items of the ADAS-Cog questionnaire: items 1, 4, 7, 8 and 9 for memory, items 2, 5, 10, 11 and 12 for language, items 3 and 6 for praxis, and item 13 for concentration.

Regional FDG-PET SUVR were extracted using the second version of the Automated Anatomical Atlas^52,53^ (see http://www.gin.cnrs.fr/fr/outils/aal-aal2/) (AAL2) with 120 regions covering the cortex and the main subcortical structures, using the open-source community software Clinica^54^ (see http://clinica.run/doc/Pipelines/PET_Volume). The software performs intra-subject registration of the FDG-PET image into the space of the subject’s T1-weighted MRI image using Statistical Parametric Mapping^55^ (see www.fil.ion.ucl.ac.uk/spm/ (SPM) software (version 12)). The PET image is then spatially normalised into MNI space using DARTEL deformation model of SPM, and its intensities normalised using the average uptake value in the pons as reference region. The SUVR map is obtained by averaging resulting intensities in each region of the atlas^56^.

The MRI images were first processed independently with the cross-sectional pipeline of the FreeSurfer^57,58^ (see https://surfer.nmr.mgh.harvard.edu software (version 5.3.0)). The longitudinal FressSurfer pipeline is then used to create subject-specific templates from the successive data of each subject and refine image segmentations^59^. These segmented images are used then to extract a cortical thickness map, and a mesh of the left and right hippocampus.

We used the cortical surface mesh projected onto the average space called FSaverage with 163,842 vertices. For dimensionality reduction purposes, we then

- inflate the FSAverage mesh to a sphere using FreeSurfer, on which 3658 vertices (called patch-nodes) are selected to map the whole sphere uniformly,
- associate each vertex to its closest patch-node, resulting in a parcellation of the cortical mesh into 3658 patches that are uniformly distributed over the surface, where a patch contains on average 44 vertices,
- compute the average value of the cortical thickness in each patch.

We also aligned the skull-stripped images with an affine 12-degrees-of-freedom transformation onto the Colin27 template brain (see http://www.bic.mni.mcgill.ca/ServicesAtlases/Colin27, using the FSL 5.0 softwarecitewoolrich2009bayesian (see https://fsl.fmrib.ox.ac.uk/fsl/fslwiki/). Mesh representations of the geometry of the left and right hippocampus result from the following steps:

- the volumetric segmentations of the hippocampi obtained by FreeSurfer are transformed into meshes using the aseg2srf software( see https://brainder.org (version of July 2009)),
- the resulting meshes are decimated by a 88% factor using Paraview, 5.4.1^60^ (see www.paraview.org),
- then aligned using the previously-computed global affine transformation estimated with the FSL software,
- residual pose differences among subjects are then removed by rigidly aligning the meshes from the baseline image of each subject to the corresponding hippocampus mesh in the Colin27 atlas image, this transformation with 6 degrees of freedom being computed with the GMMReg software^61^ (see https://github.com/bing-jian/gmmreg (version of July 2008)),
- the same transformation is eventually used to align the meshes from the follow-up images of the same subject.

### Disease course mapping

Disease course mapping models changes in image and clinical data during disease progression. Its core aspect is the construction a long-term normative scenario of progression by normalizing and mapping subject’s data to different portions of a common trajectory. This mapping includes notably the estimation of an Alzheimer Age at each visit of each patient.

We consider first the simple case where one observes only two biomarkers at each visit of each patient. As illustrated in the Figure 1 (top row), the idealized model of progression is mapped to the individual data of a subject using three parameters:

- time-shift $\tau _i$, the temporal onset of disease progression for all biomarkers in subject i; $\tau _i$ is negative for earlier-than-average progression and positive for later-than-average progression,
- acceleration $\alpha _i$, the change in the rate of progression of subject i; $\alpha _i=1$ means that, for that subject, all the biomarker trajectories match the idealized curves after adjusting the timing with $\tau _i$ and $\omega _i$. When $\alpha _i>1$, it takes less time for the same changes to occur, e.g. $\alpha _i=2$ means that it will twice as fast as average for that subject to undergo a given change in a biomarker trajectory,
- intermarker spacing $\omega _{ik}$, the temporal ordering of biomarker k with respect to other biomarkers for patient i. The sign of $\omega _{ik}$ indicates whether a single feature in a particular subject exhibits earlier or later changes relative to other features (after normalizing the age at onset with $\tau _i$ and pace of progression with $\alpha _i$: $\omega _{ik}<0$ means earlier degradation, and $\omega _{ik}>0$ means later.

For the model to be identifiable, the effect of the intermarker spacing on the curves should not overlap with the time-shift $\tau _i$. If the two logistic curves are parallel, one intermarker spacing should be the opposite to the other one. If the two logistic curves are not parallel, a weighted sum of the inter-marker spacing should sum up to zero, where the weights depend on the parameters of the logistics. The consequence is that for *N* biomarkers, there is only $N-1$ independent intermarker spacing^11,12^.

To go further, we now plot one biomarker versus the other one in Figure 1 (bottom row). The logistic progression of the two biomarkers forms a curve in the unit square starting at point of coordinate (0, 0) when both biomarkers are normal and ending at point of coordinate (1, 1) when both biomarkers reach their maximal value. One point represents the two biomarkers of one patient at one time-point. Time is implicit in this representation.

The effect of the time-shift $\tau$ and acceleration $\alpha$ is to slide the points along the curve so that the actual age of the subject at each visit moves to the corresponding Alzheimer Age (i.e. the time index of the points closest to the curve). These parameters change the parametric representation of the curve, not the curve itself. By contrast, the effect of the interspacing marker is to translate the subject’s curve in the direction given by the vector $\omega = (\omega _1,\omega _2)$ to fit the reference population curve. We impose the vector $\omega$ to be orthogonal to the velocity of the curve to ensure that the effect of the intermarker spacing does not overlap with that of $\tau$ and $\alpha$. This condition amounts to imposing that a weighted average of the coordinates of the intermarker spacing equal zero.

This representation of the method permits now its generalisation to a large class of data. In the previous example, the unit square is a particular case of a Riemannian manifold, a mathematical space that generalise the usual Euclidean plane. The central curve is a geodesic on this manifold, namely a curve of minimal energy that is fully determined by its velocity at a given point and time. The change in the parametric representation of the curve with the time-shift $\tau$ and acceleration $\alpha$ applies to any types of curves. The translation in the direction of $\omega$ is a generalisation of the usual translation that we have introduced and called “Exp-parallelisation”^11,12^.

Therefore, the exact same construction applies for any data which may be represented on a Riemannian manifold. One must only specify the Riemmanian manifold, in particular its metric which determines the parametric form of the geodesics. Then, the normative scenario of progression is assumed to be a geodesics in this manifold. Subjects are assumed to follow a piece of curve that derive from the normative curve by a change in the parametric representation and an Exp-parallelisation. This construction defines a spatiotemporal coordinate system around the reference curve that is used to position the subjects trajectories.

We consider cortical thickness measurements and images as a series of biomarkers (one per region, voxel or vertex). We define the Riemannian metric such that each biomarker follow a straight line and the intercept and slopes of the straight lines vary smoothly across neighbourhing regions, voxels or vertices^62,63^. We consider changes in the shape of the hippocampus are due to a smooth deformation of the surface called diffeomorphisms^64,65^.

The central geodesics, namely the idealized set of logistics in the two biomarkers example, serves as a reference pattern of progression. Our fitting to this curve requires simultaneous estimation of the individual parameters ($\tau _i$, $\alpha _i$, and $\omega _{ik}$ of each subject in a longitudinal setting. Each subject has a single $\tau$, a single $\alpha$ and a multivariate $\omega$ (one per biomarker in the biomarker example, in other examples the size of $\omega$ is the dimension of the considered Riemmanian manofold). We estimate the parameters of the geodesics, namely the shape and position of the logistic curves in the biomarker example, so that mean value of the $\tau _i$, $\log (\alpha _i)$ and each of the $\omega _{ij}$ across all subjects is zero. The resulting trajectory represents the progression of the average subject in the studied population. We iteratively perform joint estimations of the population and individual parameters to minimize the residual variance.

The disease course map results from the calibration of the method on a longitudinal data with at least two time-points per subject. The disease course map includes the estimated normative scenario of change and the empirical distribution of the individual parameters $\tau$, $\alpha$ and $\omega$.

Once the disease course map is calibrated, one can position new subjects in the map and predict their future progression. We use the baseline data of the subjects, with additional follow-up if available, to estimate the individual parameters for the subjects and transform the normative scenario of changes to match empirical data (see Figure 1). One the one hand, the individual parameters position subjects among themselves. One may then test for example if some genetic factors favor early or late disease onset, fast or slow progression, or specific patterns of progression such as a specific ordering in the decline of cognitive functions. One the other hand, the personalized scenario provides a prediction of the future values of the subject’s data at any time-point in the future. Eventually, one may simulate random transformation of the reference scenario to synthesise virtual patients developing the disease.

### A non linear mixed-effects model

All in one, this construction presented above defines a non-linear mixed-effects statistical model. We denote $\gamma _0(t)$ the population geodesic curve where *t* is the Alzheimer age, $\eta ^{w_i}[\gamma _0](t)$ the parallel shift of the population curve in the direction $w_i$, and $\psi _i(t) = \alpha _i(t-t_0-\tau _i) + t_0$ the time-reparameterisation function, called time-warp, determined by the time-shift $\tau _i$ and acceleration $\alpha _i$. It maps the actual age of the subject to its Alzheimer age. The value of $\gamma _0(t)$ at time *t* results from the integration of a second-order ordinary differential equation. It is therefore a function of its initial conditions: an initial point $p_0$ and a velocity $v_0$ at a given time-point $t_0$.

The j-th observation of the i-th subject, denoted $y_{ij}$ acquired at actual age $t_{ij}$ is then assumed to be derived from the population curve by $y_{ij} = \eta ^{w_i}[\gamma _0](\psi _i(t_{ij})) + \varepsilon _{ij}$ for the $\varepsilon _{ij}$ is a random noise.

The model may be written in short as $y_{ij} = f(\theta ,z_i,t_{ij}) + \varepsilon _{ij}$, for *f* a non-linear function that is specific to each data type, $\theta$ the vector containing the fixed-effects $p_0,v_0,t_0$, the variance of the random-effects and the variance of the noise, and $z_i$ the vector of random effects: acceleration factors, time-shifts and space-shifts. We add priors on the coordinates of the vector $\theta$ in a Bayesian setting. When *t* is varied, the curve $f(\theta ,z_i,t)$ represents the subject-specific trajectory at any time *t*.

We now consider four statistical tasks:

- **calibration:** given the longitudinal data set $\{y_{ij},t_{ij}\}_{i=1,\ldots ,N,j=1,\ldots ,N_i}$, we find the value of parameters $\theta$ that maximises the joint likelihood $p(\{y_{ij}\}_{ij}, \theta ) = p(\{y_{ij}\}_{ij}| \theta )p(\theta )$. The optimal value ${{\hat{\theta }}}$ fully specifies the disease course map;
- **personalisation:** for the optimal value of the parameter ${{\hat{\theta }}}$, we personalise the model to the repeated data of a given subject (either a training subject, or a test subject in a cross-validation setting) $\{y_{test,j},t_{test,j}\}_{j=1,\ldots ,N_{test}}$ by finding the optimal value of the random-effect ${{\hat{z}}}$ that maximises the conditional likelihood $p(\{y_{test,j}\}_{j}, z \vert {{\hat{\theta }}})$. The resulting $f({{\hat{\theta }}}, {{\hat{z}}}, t_{test,j})$ is called the **reconstruction** of the data $y_{test,j}$ and its difference with the true data $y_{test,j}$ is called the reconstruction error;
- **prediction:** given a test subject with $N_{test}$ observations, we personalize the model using only the first $N_{past}$ ($<N_{test}$) observations to estimate ${{\hat{z}}}$, and then predict the future data after $N_{past}$ by extrapolating the trajectory $f({{\hat{\theta }}}, {{\hat{z}}}, t_{test,j})$, and measure the prediction error between the predicted and true (hidden) data.
- **simulation:** for the optimal value of the parameter ${{\hat{\theta }}}$, we simulate random-effects *z* and generate synthetic data *y* at any user-defined time-point *t* by computing $y = f({{\hat{\theta }}},z,t)$ and adding noise.

We use a stochastic approximation of the Expectation-Minimisation algorithm^66–68^ for calibration, gradient-descent based method or Powell’s method for personalisation, and kernel density estimation together with dimension reduction for simulation.

We computed Student t-test between cofactors and estimates of the random effects using the False discovery rate method for the correction for multiple comparisons. A graph of conditional correlations is obtained using an exploration and selection method adapted in the high-dimensional low sample size setting^69^.

### Estimation of measurement uncertainty

In the ADNI protocol^70,71^, most MRI sessions consist of a pair of test and re-test MRI, namely two scans performed on the same day one immediately after the other one. For 1,841 out of 1,993 MRI sessions, we measure therefore the differences between the MRI derived data (hippocampus meshes and cortical thickness maps) when using the test or the re-test image in the processing pipeline describe above. These differences give an empirical distribution of the noise due to variations in image acquisition and processing.

For PET derived data, we use the baseline and follow-up scans of stable cognitively normal and amyloid negative subjects in ADNI, as a proxy to test / re-test data (125 subjects, 244 visits with a follow-up time of 18 months).

Test / re-test studies have shown a that the MMSE, which scales from 0 to 30, is subject to a difference between two sessions, whose standard deviation ranges from 1.3 for a one-month interval^39^ up to 1.82 for a 1.5 year long interval^72^, thus representing a standard deviation of 4.3 to 6%. Another study^73^ measured the former ADAS-Cog that scales between 0 and 70 three times at a 2-week interval, with an agreement between raters. The inter-ratter standard deviation is of 9.64 between the first and second test, and of 6.79 between the second and third test. The intra-rater standard deviation is of 8.16 between the first and third visit. This corresponds to a standard deviation ranging from 9.7% to 13.8%. On average, we consider such neuro-psychological assessments to have a zero-mean Gaussian distribution of noise with standard deviation of 7%.

## Supplementary Information

Supplementary Information.
