# Supplementary material for: AD Course Map charts Alzheimer’s disease progression
Source: Sci Rep. 2021 Apr 13;11:8020. doi: 10.1038/s41598-021-87434-1 (PMC8044144; doi:10.1038/s41598-021-87434-1)
Supplement: Supplementary file 1 — Supplementary Information. [file 41598_2021_87434_MOESM1_ESM.pdf]

AD Course Map charts Alzheimer's disease progression

I. Koval, A. Bône, M. Louis, Th. Lartigue, S. Bottani, A. Marcoux, J. Samper-González, N. Burgos, B. Charlier, A. Bertrand, S. Epelbaum, O. Colliot, S. Allassonnière, S. Durrleman

Supplementary Information

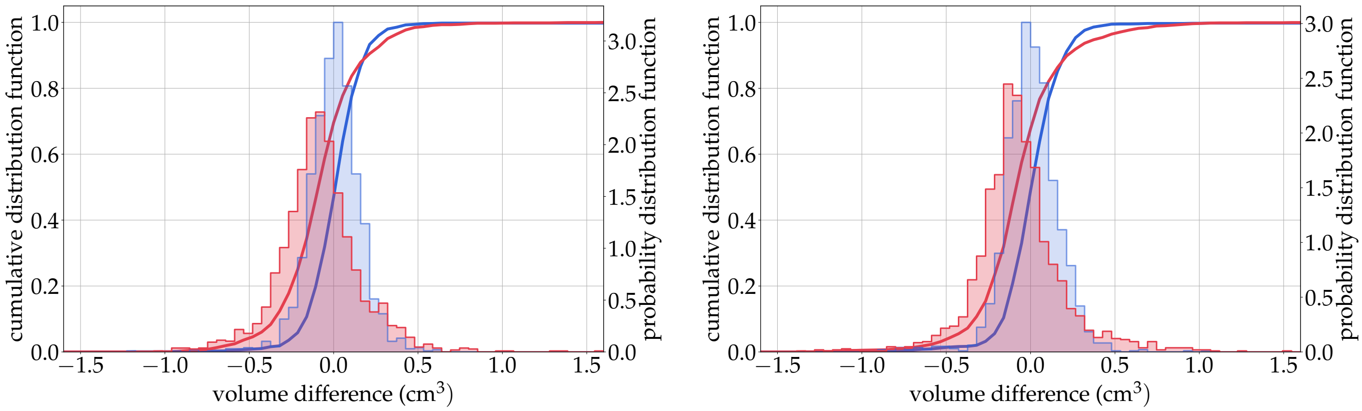

Supplementary Figure S1. Reconstruction errors for the hippocampus meshes measured as volumes. Superimposition of the distribution of the reconstruction errors (in red) and test / re-test differences (in blue) measured as volumes for the left and right hippocampus (left and right panel respectively). Reconstructed meshes are smoother than observations, thus with a smaller volume than the observations which often show spikes pointing outward of the surface. Mean reconstruction error is of -84.5 mm^3 for the left hippocampus and -67.3 mm^3 for the right hippocampus.

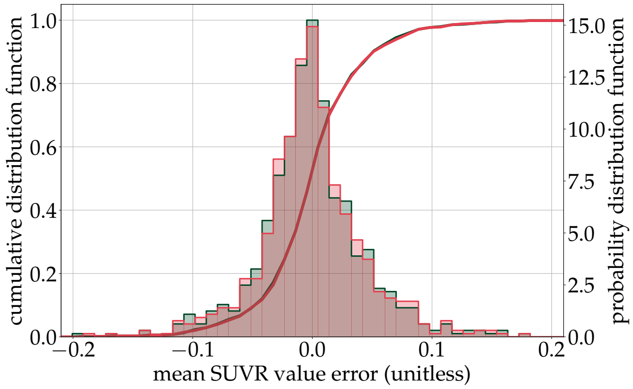

(a) FDG-PET SUVR values. The mean error is of $1.0 \times 10^{-4} \pm 0.044$ (red), and $-1.3 \times 10^{-4} \pm 0.044$ (green).

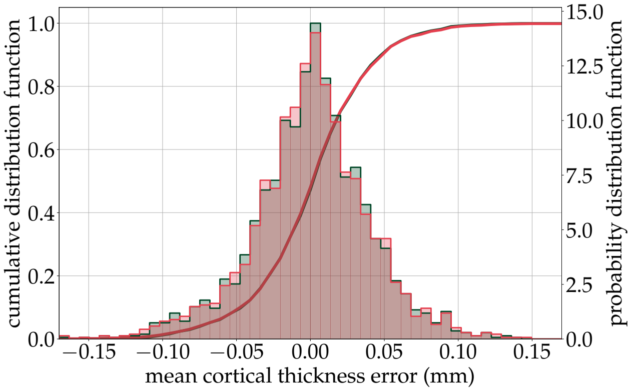

(b) Mean cortical thickness. The mean error is of $5.8 \times 10^{-4} \pm 0.040 mm$ (red) and $6.1 \times 10^{-4} \pm 0.040 mm$ (green).

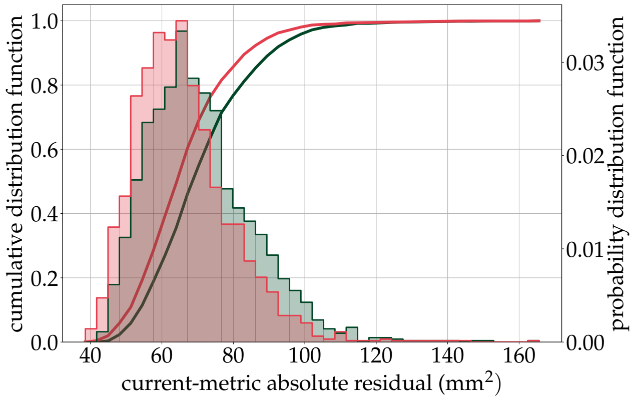

(c) Left hippocampus. The mean error is $66.0 \pm 13.6 mm^2$ (red), and $70.7 \pm 14.9 mm^2$ (green).

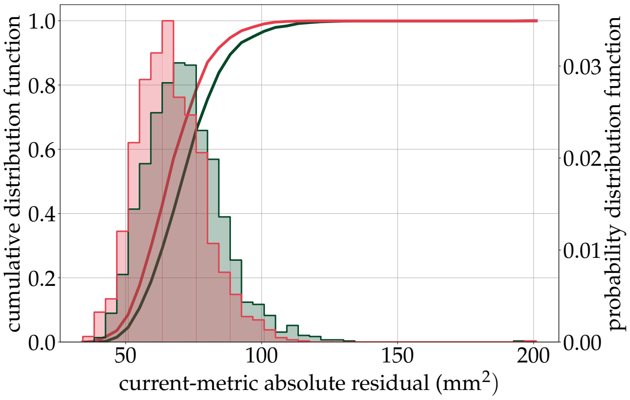

(d) Right hippocampus. The mean error is $66.6 \pm 12.8 mm^2$ (red), and $71.7 \pm 14.0 mm^2$ (green).

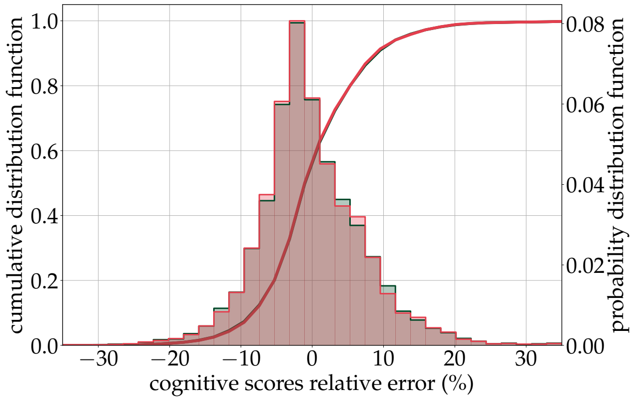

(e) Neuro-psychological assessments. The mean error is $-0.19 \pm 7.5\%$ (red), and $-0.14 \pm 7.5\%$ (green).

Supplementary Figure S2. Generalisation error to unseen data. The distribution of reconstruction errors when calibration and personalisation are done on the whole data set (in red, as in Fig. 2) is superimposed with the one estimated in the cross-validation procedure (in green).

<table>
<tr><th>Modality</th><th>Time-shift</th><th>acceleration factor</th></tr>
<tr><td>FDG-PET SUVR</td><td>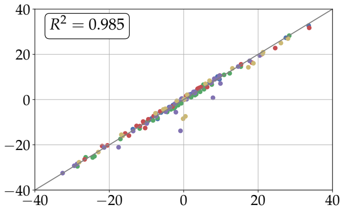
</td><td>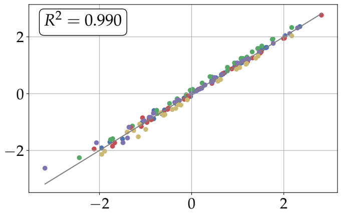
</td></tr>
<tr><td>Cortical thickness</td><td>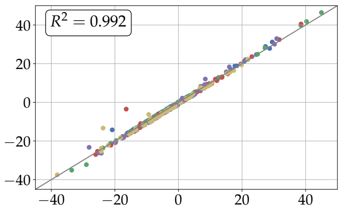
</td><td>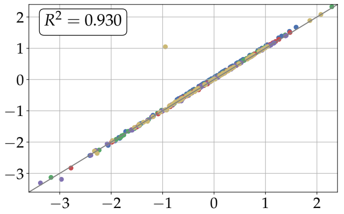
</td></tr>
<tr><td>Right hippocampus</td><td>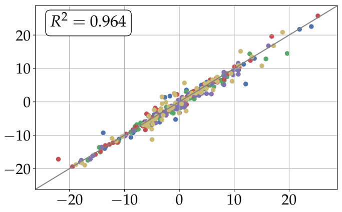
</td><td>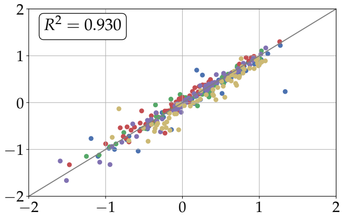
</td></tr>
<tr><td>Left hippocampus</td><td>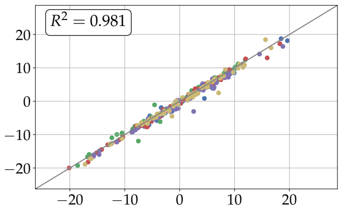
</td><td>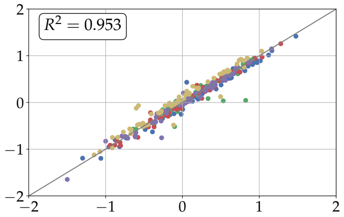
</td></tr>
<tr><td>Neuro-psychological scores</td><td>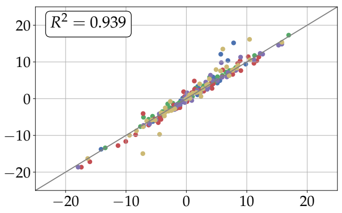
</td><td>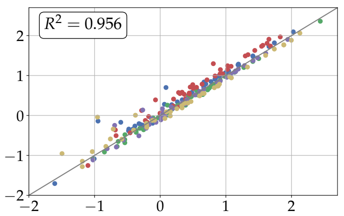
</td></tr>
</table>

Supplementary Figure S3. Robustness of model calibration and personalisation. Estimated time-shifts and acceleration factors when the individual belongs to the training set (x-axis) or to the test-set (y-axis). The five colors correspond to the folds the individuals belong to.

Modality (unit)	Mean Error (± std)		Mean Absolute Error (± std)	
	Reconstruction	Estimated measurement uncertainty	Reconstruction	Estimated measurement uncertainty
FDG-PET (no unit)	$1.1 \times -10^{-4}(\pm 0.10)$	$-3.0 \times 10^{-3}(\pm 0.095)$	$7.6(\pm 6.5) \times 10^{-2}$	$6.8(\pm 9.4) \times 10^{-2}$
Cortical thickness (mm)	$5.8 \times 10^{-4}(\pm 0.44)$	$-1.1 \times 10^{-3}(\pm 0.28)$	$0.35(\pm 0.28)$	$0.19(\pm 0.20)$
Right hippocampus (mm^2)	$69.8(\pm 15.0)$	$85.2(\pm 40.1)$	$69.8(\pm 15.0)$	$85.2(\pm 40.1)$
Left hippocampus (mm^2)	$68.5(\pm 15.9)$	$83.2(\pm 36.0)$	$68.5(\pm 15.9)$	$83.2(\pm 36.0)$
Cognitive scores (no unit)	$-2.2 \times 10^{-3}(\pm 0.075)$	$0(\pm 0.070)$	$5.5(\pm 5.0) \times 10^{-2}$	$5.6(\pm 4.2) \times 10^{-2}$

Supplementary Table S1. Comparison between the statistics of the reconstruction errors and the ones of the distribution of the measurement uncertainty. For hippocampus meshes, differences are measured by the norm of a vector, namely a positive number, so that errors and absolute errors coincide. For cognitive scores, the estimated measurements noise are computed based on the hypothesis of a centered Gaussian distribution with 7% standard deviation derived from the literature (see Methods).

Modality	Parameters	All data	Cross-validation
FDG-PET images	σ (no units)	0.101	0.101 ($\pm$ 0.001)
	t_0 (years)	75.5	74.9 ($\pm$ 0.9)
	σ_τ (years)	11.9	11.5 ($\pm$ 0.3)
	σ_ξ (no units)	1.30	1.28 ($\pm$ 0.03)
Cortical thickness	σ (mm)	0.442	0.442 ($\pm$ 0.001)
	t_0 (years)	82.0	82.7 ($\pm$ 0.7)
	σ_τ (years)	16.9	18.2 ($\pm$ 0.7)
	σ_ξ (no units)	0.99	1.03 ($\pm$ 0.02)
Right hippocampus	σ (mm^2)	2.49	2.60 ($\pm$ 0.03)
	t_0 (years)	76.2	75.7 ($\pm$ 0.3)
	σ_τ (years)	9.15	10.04 ($\pm$ 0.66)
	σ_ξ (no units)	0.71	0.78 ($\pm$ 0.03)
Left hippocampus	σ (mm^2)	2.67	2.74 ($\pm$ 0.04)
	t_0 (years)	76.3	76.3 ($\pm$ 0.3)
	σ_τ (years)	8.53	9.09 ($\pm$ 0.50)
	σ_ξ (no units)	0.66	0.68 ($\pm$ 0.03)
Cognitive scores	σ (no units)	0.081	0.081 ($\pm$ 0.001)
	t_0 (years)	71.5	72.4 ($\pm$ 0.8)
	σ_τ (years)	7.29	7.36 ($\pm$ 0.25)
	σ_ξ (no units)	1.07	1.11 ($\pm$ 0.11)

Supplementary Table S2. Fixed-effects estimates using calibration on the whole data set (first column) and in a five fold cross-validation setting (second column) where mean and standard deviations of the five estimates are shown. Similarly, the delay between impairment of memory and the other cognitive functions is of 9.4 ± 1.6 yrs for concentration (9.6 yrs using all data), 19.9 ± 2.0 yrs for praxis (19.4 yrs using all data), 23.3 ± 2.6 yrs for language (22.7 yrs using all data)

	ADAS & MMSE	PET	MRI
Number of subjects	223	157	322
Number of visits	1,235	690	1,993
Average number of visits per subject ($\pm$ std)	5.5 ($\pm$1.1)	4.4 ($\pm$ 2.1)	5.8 ($\pm$ 2.4)
Average age ($\pm$ std)	76.2 ($\pm$ 6.9)	74.0 ($\pm$ 7.2)	74.0 ($\pm$ 6.7)
Sex ratio (F/M in %)	39.0 / 61.0	41.8 / 58.2	41.2 / 58.8
Amyloid status (+/-/unknown in %)	65.5 / 7.2 / 27.3	77.4 / 7.3 / 15.3	73.2 / 7.1 / 19.7
APOE carriership (%)	62.8	64.2	65.2
Education (mean $\pm$ std, in years)	15.8 ($\pm$ 2.8)	15.8 ($\pm$ 2.7)	15.9 ($\pm$ 2.8)
Marital status (married/not married in %)	81.2 / 18.8	82.3 / 17.7	80.9 / 19.1

Supplementary Table S3. Summary statistics of the subject subsets for each data type
